# Genetic Characterization of *Toxoplasma gondii* Isolates and Toxoplasmosis Seroprevalence in Stray Cats of İzmir, Turkey

**DOI:** 10.1371/journal.pone.0104930

**Published:** 2014-08-15

**Authors:** Hüseyin Can, Mert Döşkaya, Daniel Ajzenberg, H. Gökhan Özdemir, Ayşe Caner, Sultan Gülce İz, Aysu Değirmenci Döşkaya, Esra Atalay, Çağdaş Çetinkaya, Saygun Ürgen, Sabire Karaçalı, Cemal Ün, Marie-Laure Dardé, Yüksel Gürüz

**Affiliations:** 1 Department of Molecular Biology, Ege University Faculty of Sciences, Bornova/İzmir, Turkey; 2 Department of Parasitology, Ege University Medical School, Bornova/İzmir, Turkey; 3 Centre National de Référence (CNR) Toxoplasmose/*Toxoplasma* Biological Resource Center (BRC), Centre Hospitalier-Universitaire Dupuytren, Limoges, France and INSERM UMR 1094, Neuroépidémiologie Tropicale, Laboratoire de Parasitologie-Mycologie, Faculté de Médecine, Université de Limoges, Limoges, France; 4 Department of Veterinary Affairs, Municipality of İzmir, Turkey; 5 Department of Bioengineering, Ege University Faculty of Engineering, Bornova/İzmir, Turkey; Obihiro University of Agriculture and Veterinary Medicine, Japan

## Abstract

Currently, some *Toxoplasma gondii* genotypes are being associated with serious clinical presentations. A recent report showing the Africa 1 genotype in two local congenital toxoplasmosis cases acquired in Turkey formed the basis of this study because atypical Africa 1 genotype is most frequently detected in animals and patients from sub-Saharan Africa. Since stray cats are considered as the linkage between wild life and urban life in *T. gondii* transmission, the present study aimed to isolate and characterize *T. gondii* strains circulating in stray cats of İzmir (Western Turkey). A secondary objective was to determine toxoplasmosis seroprevalence in this cat population. Tissues obtained from 100 deceased stray cats were bioassayed and isolated strains were genotyped using 15 microsatellite markers. In addition, toxoplasmosis seroprevalence was analyzed in 1121 cat sera collected from several large veterinary clinics in İzmir. Among the 22 isolates, 19 were Type II (86.3%), two were Type III (9%) and one was Africa 1 genotype (4.5%). The overall seropositivity rates in cats were 42–48% and 33.4–34.4% according to IFA and ELISA, respectively. Seroprevalence in deceased cats was significantly higher than in healthy cats (*P* = 0.0033). Finding both the major clonal Type II lineage together with the Type III lineage also found in Middle East, and an atypical genotype, Africa 1 appears consistent with the specific geographic location of Turkey between three continents and raises the possibility of transportation of these strains between continents through trade routes or long distance migratory birds. In addition, the first large study of toxoplasma seroprevalence in a stray cat population was also reported. The relatively high seropositivity rates and the variety of *T. gondii* genotypes confirm the local stray cat population as a risk factor for human toxoplasmosis in İzmir.

## Introduction

*Toxoplasma gondii* is a protozoon parasite that causes serious clinical problems. Currently, *T. gondii* genotypes are being associated with various clinical presentations. *T. gondii* strains have been classified by genetic polymorphism into three major clonal lineages and other additional lineages, as well as atypical and recombinant strains [1]–[3].

Particular genotypes have been shown to have different geographical distribution. In Europe, type II is frequently isolated from human and animals [4]. In sub-Saharan Africa, non-archetypal genotypes named Africa 1, 2, and 3 have been isolated from humans and domestic animals in addition to three major lineages [2], [5], [6]. In North Africa, the Middle East and the Arabic peninsula, type II and III strains are prevalent [7]–[9]. In Asia, the three major clonal lineages have been identified in addition to the predominant genotype, Chinese 1 [10], [11], [12], [13]. In North and Central America, the three major lineages and recombinant strains exist but in South America, there is high diversity within and between *T. gondii* populations [14], [15], [16].

An interesting finding, i.e. the detection of Africa 1 genotype in two local congenital toxoplasmosis cases whose mothers lived in Turkey has formed the basis of this study because atypical Africa 1 genotype is frequently detected in animals and immunocompromised patients from sub-Saharan Africa [6], [17]. This finding raised questions about the source of *T. gondii* strains in Turkey. In particular, whether there are more atypical genotypes in Turkey, and whether all three major clonal lineages are present.

Due to its specific geographic location between Africa, Europe and Asia, Turkey is like a bridge on the historic trade routes. *T. gondii* strains may have been transferred between these continents via cats that were kept in trade ships or caravans for petting and rodent hunting purposes [17]. Another possible way of intercontinental transport may be through long distance migratory birds. Thus, strains detected mainly in Europe, Africa and Asia may also be detected in Anatolia, or vice versa.

**Figure 1:**
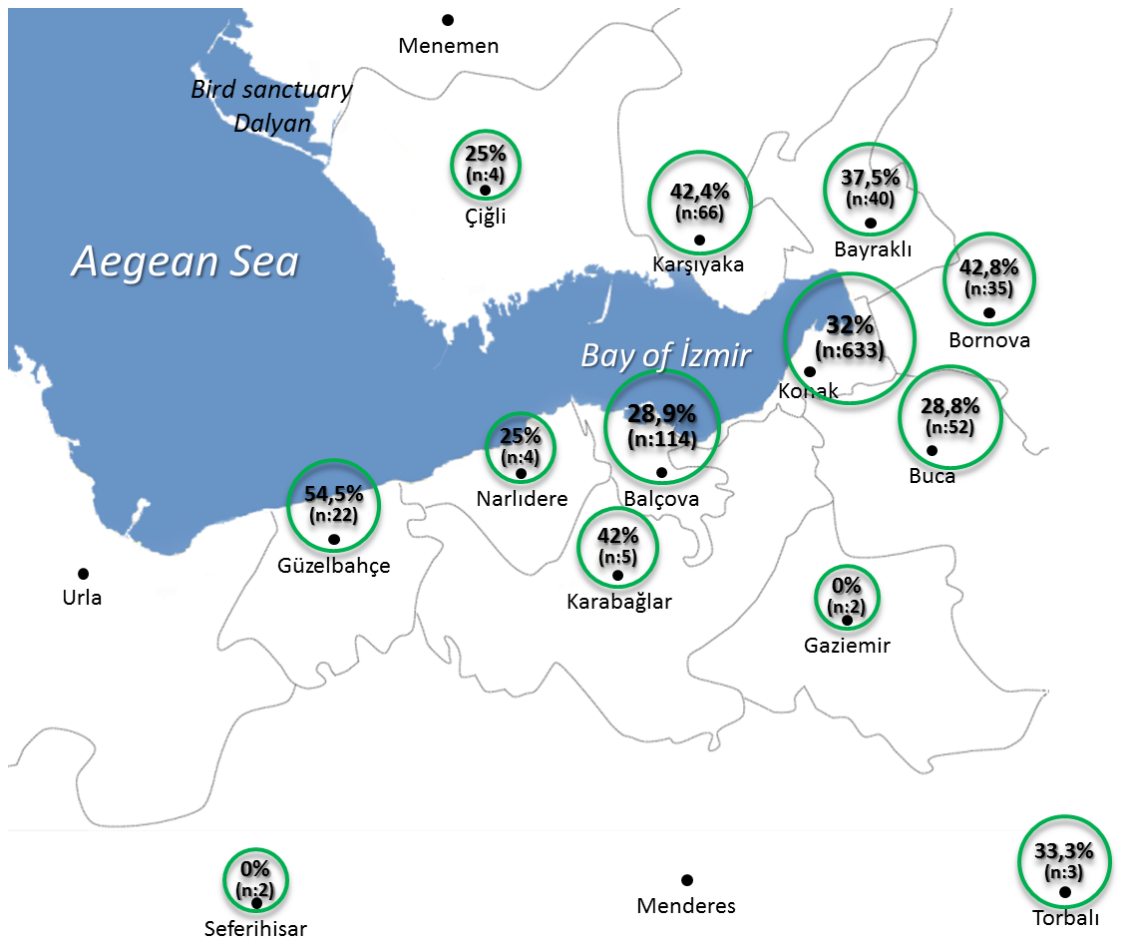
The geographic distribution of seropositivity rates in serologically coherent stray cat sera from various locations of İzmir.

İzmir is the third biggest city in Turkey located close to the Western Anatolian historic trade routes and has a huge wild life park with bird sanctuary (Figure 1). In the complex life cycle of *T. gondii*, sexual reproduction occurs in the definitive host, felines, and stray cats are considered as the main linkage between wild life and urban life in *T. gondii* transmission [18]. Therefore, the present study aimed to isolate and characterize *T. gondii* strains genetically and determine toxoplasmosis seroprevalence in stray cats of İzmir.

## Materials and Methods

### 1. Ethics statement

All experiments were performed under the instructions and approval of the Institutional Animal Care and Use Committee (IACUC) of Ege University for animal ethical norms (Permit number: 2010-72). Animals were housed under standard and suitable conditions.

6–8 week old female *Swiss outbred* mice were obtained from the Bornova Veterinary Control Institute Animal Production Facility.

Stray cat samples were provided by the Veterinary Clinic, Municipality of İzmir, Turkey. The medical care of healthy and deceased stray cats officially belong to the Municipality of İzmir. Accordingly, the owners of the “Healthy cats brought to Veterinary Clinic of Municipality of İzmir, for sterilization purposes” gave permission for the animals to be used in this study.

### 2. Cats and sample collection

The stray cats were brought to the Veterinary Clinic from 16 different locations of İzmir (Balçova, Bayraklı, Konak, Buca, Bornova, Karşıyaka, Çiğli, Gaziemir, Güzelbahçe, Torbalı, Narlıdere, Özdere, Karabağlar, Balçova, Konak and Karşıyaka) (Figure 1).

Tissue (brain and heart) samples were collected from deceased cats (n: 100). Sera were collected from live cats (n: 1021) and exudate from coagulated blood in heart was collected from deceased cats. Live cats were brought to Veterinary Clinic for sterilization purposes from Konak (n:659), Balçova (n:122), Buca (n:55), Bornova (n:39), Bayraklı (n:44), Karabağlar (n:55), Güzelbahçe (n:25), Çiğli (n:5), Karşıyaka (n:5), Narlıdere (n:5), Torbalı (n:3), Gaziemir (n:2), and Seferihisar (n:2). Deceased cats were those brought to the Veterinary Clinics due to several health problems from Konak (n:28), Balçova (n:4), Karşıyaka (n:67) and Bayraklı (n:1) (Figure 1). Tissue and sera samples were collected from cats after they died in the clinic.

### 3. Bioassay in mice

The tissue homogenates were prepared from the brain and heart tissue of the cats as described [6]. Briefly, 0.9% NaCl was added to tissues (10 g tissue/125 ml 0.9% NaCl) and homogenized using a blender (Waring, USA). Thereafter, trypsin was added to the homogenate (0.5 g trypsin/10 g tissue) and incubated at 37°C for 75 min in an incubator shaker (New Brunswick, USA). After incubation, the homogenate was filtered through sterile two layered gauze and centrifuged at 910×g. After discarding supernatant, the pellet was washed two more times with 0.9% NaCl. After the last centrifugation the supernatant was discarded, the pellet was homogenized with ∼10 ml 0.9% NaCl and a 500 µl aliquot was kept for DNA extraction. Penicillin (40 U/ml)/Streptomycin (40 µg/ml) and Gentamycin (40 µg/ml) were added to the remaining homogenate, incubated at 4°C overnight and ∼700 µl homogenate was inoculated intraperitoneally to mice (3–6 mice/group). After 40 days, the mice were sacrificed, and thereafter *T. gondii* tissue cysts and DNA were investigated in brain homogenates of mice using phase contrast microscopy (Nikon, USA) and Real Time PCR, respectively. Live strains were cryopreserved with RPMI containing 10% FCS and 10% DMSO.

#### 3.1. Real Time PCR

*T. gondii* AF146527 gene was investigated by Real Time PCR as described [19]. Briefly, isolation of DNA from the cat tissue or mouse brain homogenate was performed by QIAamp DNA mini kit (Qiagen, USA) according to the manufacturer's protocol. During Real Time PCR, the primers used for amplifying 134 bp AF146527 gene fragment were 5′-AGGCGAGGGTGAGGATGA-3′ (18 nt, TOX-SE forward primer) and 5′-TCGTCTCGTCTGGATCGCAT-3′ (20 nt, TOX-AS reverse primer). The hybridization probes were 5′-GCCGGAAACATCTTCTCCCTCTCC-3′-FL (24 nt, TOX FLU, labeled at the 3′ end with fluorescein) and 5′-640-CTCTCGTCGCTTCCCAACCACG-3′ (22 nt, TOX LCR labeled at the 5′ end with LC-Red 640) (Metabion, Germany). The parasite quantification and melting curve analysis was performed by 1.2 LightCycler Real Time instrument using LightCycler software, Version 3.5 according to the manufacturers protocol (Roche). 20 µl final volume PCR reaction included 1x LightCycler Fast Start DNA Master HybProbe mix (Final concentration of MgCl_2_ is adjusted to 5 mM) (Roche), 5 µl purified sample DNA template or controls. The PCR amplification reactions were performed by the following calculated protocol: 10 min initial denaturation step at 95°C, followed by 50 cycles of 5 seconds at 95°C, 10 seconds at 60°C, and 15 seconds at 72°C. As positive controls, *T. gondii* genomic DNA serially 10-fold diluted ranging from 10^6^ to 10^1^ parasites per µl and one negative control prepared by replacing template DNA with distilled water were used. Melting curve analysis was performed using the following calculated protocol: 20 s denaturation step at 95°C with temperature transition rate 20°C/s followed by 20 s annealing step at 40°C with temperature transition rate 20°C/s and extension step gradually increasing temperature to 85°C with temperature transition rate 0.2°C/s.

### 4. Genotyping analysis

For the microsatellite analysis of strains, single multiplex PCR assay detecting 15 microsatellite markers (*TUB-2, W35, TgM-A, B18, B17, M33, IV.1, XI.1, M48, M102, N60, N82, AA, N61, N83*) located on 11 different chromosomes of *T. gondii* was used as described [20]. Briefly, 25 µl amplification reaction included 5 µl DNA extracted from mouse brain or cat tissues, 15 pairs of primers (5 pmol each) and 12.5 µl multiplex PCR master mix (Qiagen).The PCR amplification reaction was performed using the following calculated protocol: 15 minutes initial denaturation step at 95°C, followed by 35 cycles of 30 seconds at 94°C, 3 minutes at 61°C, and 30 seconds at 72°C, and a final extension of 30 minutes at 60°C. After amplification reaction, PCR products were diluted 1/10 (mouse brain homogenate) or 1/2 (cat tissue homogenate) in deionized formamide. Thereafter, 1 µl of diluted PCR product was mixed with 0.5 µl dye labeled DNA standard ROX 500 (Applied Biosystems) and 23.5 µl deionized formamide. Mixture was denatured for 5 min at 95°C and electrophoresed using an automatic sequencer (ABI PRISM 3130 xl; Applied Biosystems). The sizes of the microsatellites were assessed using GeneMapper analysis software (Version 4.0; Applied Biosystems). During the analysis, 17 reference strains belonging to type I (ENT, GT1), type II (Me49, PRU), and type III (NED, VEG) as well as atypical strains from Africa (DPHT, GAB3-GAL-DOM014, GAB5-GAL-DOM001, GAB3-GAL-DOM002, CCH002-NIA, and GAB2-GAL-DOM002), Turkey (Ankara and Ege-1 strains) and South America (TgCatBr5, VAND, and GUY-CAN-FAM001) were studied in parallel with strains isolated in this study [2], [6], [20]–[22].

### 5. Clustering analysis

To quantify the extent of genetic distance among strains from stray cats in Izmir, Turkey, and evaluate their position towards reference strains from different continents, Neighbor-joining trees were reconstructed from the genetic distances among individual isolates using Populations 1.2.30 (1999, Olivier Langella, CNRS UPR9034, http://bioinformatics.org/,tryphon/populations/). Trees were reconstructed using the Cavalli-Sforza and Edwards chord-distance estimator [23]. This analysis was repeated for 1000 bootstrap replicates in which loci were sampled with replacement. Unrooted trees were obtained with MEGA 6.05 software.

### 6. Serological assays

Anti-*T. gondii* IgG antibodies were investigated in 1121 cats (1021 healthy and 100 deceased cats) by IFA and *in house* ELISA. Positive/negative control sera were obtained from *T. gondii* strain isolated/unisolated cats. The presence/absence of anti-*T. gondii* IgG antibodies in these control sera were determined by a commercial ID.VET ELISA kit (ID.VET Innovative Diagnostics, France).

#### 6.1. ID.VET ELISA

During the assay, 46 cat sera were analyzed for the presence of anti-*T. gondii* IgG antibodies against P30 (SAG1) antigen according to the manufacturer's protocol (ID.VET Innovative Diagnostics). Among these 46 samples, 10 of them were collected from *T. gondii* isolate-positive cats. The remaining 36 sera were selected randomly from *T. gondii* isolate-negative cats. ID.VET ELISA was performed to determine the positive and negative sera that can be used in *in-house* ELISA and IFA.

Briefly, 100 µl of sera and kit controls at dilution of 1/10 were added to microtitre plates coated with P30 antigen, incubated for 45 min at room temperature and washed three times. Then, the wells were probed with 100 µl conjugate, incubated for 30 min at room temperature and washed three times. Bound antibodies were visualized after adding 100 µl substrate to each well. Reaction was stopped by adding 100 µl 0.5 M H_2_SO_4_ stop solution and the results were evaluated in a micro titer plate reader (Bio-Tek ELx808, USA) at 450 nm. The test results were interpreted according to the manufacturer's instructions by calculating the S/P value = (OD_sample_-OD_negative control_/OD_positive control_-OD_negative control_)×100. Serum was considered positive if the S/P value ≥50%.

#### 6.2. In house IgG ELISA

In house IgG ELISA was performed as described previously with some modifications [24], [25]. Briefly, each well of the flat bottom high binding microtiter plate (Costar, USA) was coated with 100 µl antigen suspension containing 6×10^5^/ml *T. gondii* Ankara strain tachyzoites, incubated for 1 hour at room temperature and washed three times with 200 µl PBS-T [PBS containing 0.05% (v/v) Tween 20 (pH: 7.4)]. Next, the plates were blocked (5% nonfat dry milk containing 0.05% PBS-T) for 30 min at room temperature and washed three times with PBS-T. Then, the plates were probed with 100 µl sera in duplicate at a dilution of 1/64 in blocking buffer for 1 hour and washed three times with PBS-T. Thereafter, the plates were probed with 100 µl peroxidase conjugated *anti*-cat IgG (1∶5000; Santa-Cruz, USA) diluted in PBS-T for 1 hour and washed three times with PBS-T. Bound antibodies were visualized after adding 3, 3′, 5, 5′ tetramethylbenzidine (TMB) substrate. Reaction was stopped by adding 75 µl of 2 N sulfuric acid and the results were evaluated in a micro titer plate reader (Bio-Tek ELx808, USA) at 450 nm. Serum was considered positive if the absorbance value (AV) exceeded the mean AV+2S.D.of the negative control serum samples.

#### 6.3. IgG immunofluorescence assay

Immunofluorescence assay (IFA) was performed as described with some modifications [24], [25]. Slides coated with *T. gondii* Ankara strain tachyzoites were probed with sera at serial dilutions of 1∶16, 1∶32, and 1∶64 in PBS (Ph 7.4) for 30 minutes at 37°C and washed three times with PBS. Then, slides were probed with fluorescein isothiocyanate (FITC) conjugated *anti*-cat IgG antibody (1∶250; Santa-Cruz, USA) diluted in PBS for 30 minutes at 37°C. Thereafter the slides were washed three times with PBS, examined under the immunofluorescence microscope (Olympus, USA) and serum dilutions ≥1∶16 were considered positive.

### 7. Statistical analysis

Data obtained during the study were processed using Prism 3.03 (GraphPad, San Diego, CA). A two-tailed unpaired t test with 95% confidence interval was used to determine the significance between the seropositivity rates of healthy and deceased cats.

## Results

### 1. Isolated *T. gondii* strains

*T. gondii* strains isolated from mice inoculated with cat tissues (bioassay) are called live strains. *T. gondii* strains isolated only by Real Time PCR from cat tissues are named toxoplasmic DNA extracts. Consequently, 20 live strains (20%) and 27 toxoplasmic DNA extracts (27%) were isolated. Among them, 15 strains were isolated by both bioassay and PCR, 5 of them only by bioassay and remaining 12 strains was isolated by PCR only (table 1). Overall, a total of 32 strains were isolated from 100 stray cats (32%).

**Table 1:** Microscopy, Real Time PCR, serology and genotyping results of 32 *T. gondii* strains isolated from stray cats of İzmir.

No a	Name of the isolate ^b^	Location of the isolate	Live/Toxoplasmic DNA extracts	IgG ELISA	IgG IFAT	Microscopy^ c^ (mouse brain)	Real Time PCR ^d,e^ (mouse brain) CP*_T_* value	Real Time PCR ^d,e^ (cat tissue) CP*_T_* value	Genotype
1	-	Konak	DNA extract	P	P	N	N	34.3	-
2	TgCatTr_Izmir 1	Konak	DNA extract	P	P	N	N	24.23	Type II
3	TgCatTr_Izmir 2	Balçova	live	P	P	P	21.65	N	Type II
4	TgCatTr_Izmir 3	Konak	live	P	P	P	21.53	30.80	Type II
5	TgCatTr_Izmir 4	Konak	live	N	N	P	31.04	34.95	Africa 1
6	TgCatTr_Izmir 5	Konak	live	P	P	P	25.7	N	Type II
7	TgCatTr_Izmir 6	Konak	live	P	P	P	21.59	32.08	Type II
8	TgCatTr_Izmir 7	Konak	live	P	P	P	19.84	N	Type II
9	TgCatTr_Izmir 8	Konak	DNA extract	P	P	N	N	26.55	Type II
10	TgCatTr_Izmir 9	Konak	live	P	P	P	20.56	33.68	Type II
11	TgCatTr_Izmir 10	Konak	live	P	P	P	17.15	23.47	Type III
12	TgCatTr_Izmir 11	Konak	live	P	P	P	18.94	35.49	Type II
13	-	Karşıyaka	DNA extract	P	P	N	N	34.18	-
14	TgCatTr_Izmir 12	Karşıyaka	live	P	P	P	20.38	33.12	Type II
15	TgCatTr_Izmir 13	Karşıyaka	live	P	P	P	18.31	33.72	Type II
16	TgCatTr_Izmir 14	Karşıyaka	live	P	P	P	21.94	30.92	Type III
17	-	Karşıyaka	DNA extract	P	P	N	N	33.72	-
18	-	Karşıyaka	DNA extract	P	P	N	N	33.06	-
19	-	Karşıyaka	DNA extract	P	P	N	N	35.35	-
20	-	Karşıyaka	DNA extract	P	P	N	N	34.16	-
21	TgCatTr_Izmir 15	Karşıyaka	live	P	P	P	20.16	33.85	Type II
22	TgCatTr_Izmir 16	Karşıyaka	live	P	P	P	17.89	28.98	Type II
23	TgCatTr_Izmir 17	Karşıyaka	live	P	P	P	17.24	33.03	Type II
24	-	Karşıyaka	DNA extract	P	P	N	N	32.54	-
25	TgCatTr_Izmir 18	Karşıyaka	live	P	P	P	14.46	33.78	Type II
26	TgCatTr_Izmir 19	Karşıyaka	live	P	P	P	16.52	N	Type II
27	-	Karşıyaka	DNA extract	P	P	N	N	34.75	-
28	TgCatTr_Izmir 20	Karşıyaka	live	P	P	P	16.28	32.96	Type II
29	-	Karşıyaka	DNA extract	P	P	N	N	31.93	-
30	-	Karşıyaka	DNA extract	P	P	N	N	34.47	-
31	TgCatTr_Izmir 21	Karşıyaka	live	P	P	P	22.92	N	Type II
32	TgCatTr_Izmir 22	Karşıyaka	live	P	P	P	17.71	34.89	Type II

a Isolates are numbered according to the date they have arrived to the laboratory. ^b^ The strains that couldn’t be genotyped are not named. ^c^ Microscopy detected the tissue cysts in mice brains. **^d^** Real time PCR was used to detect *T. gondii* DNA in mice brains and cat tissues. **^e^** CP*_T_* (Crossing point threshold) is the value that *T. gondii* DNA is detected. The amount of DNA is high when the value is low. N: negative; P: positive.

### 2. Microsatellite genotyping

22 strains were genotyped by microsatellite analysis and among them 19 isolates were Type II, 2 isolates were Type III and one isolate was Africa 1 genotype. Remaining 10 samples were Toxoplasma DNA extracts that were only isolated from cat tissue samples and couldn’t be genotyped due to low DNA concentration as detected by Real Time PCR (Table 1). Among the 28 samples analyzed in Konak, 11 strains were isolated (39.2%) and 10 of them were genotyped in which one of them was Africa 1, one of them was Type III and remaining eight were Type II strains. In Karşıyaka, 67 samples were analyzed, 20 strains isolated (29.8%) and 11 of them were genotyped in which 10 of them were Type II and one of them was Type III. Among the four samples from Balçova, only one Type II strain was isolated. The isolated and genotyped 22 strains were designated TgCatTr_Izmir 1 to 22.

**Figure 2:**
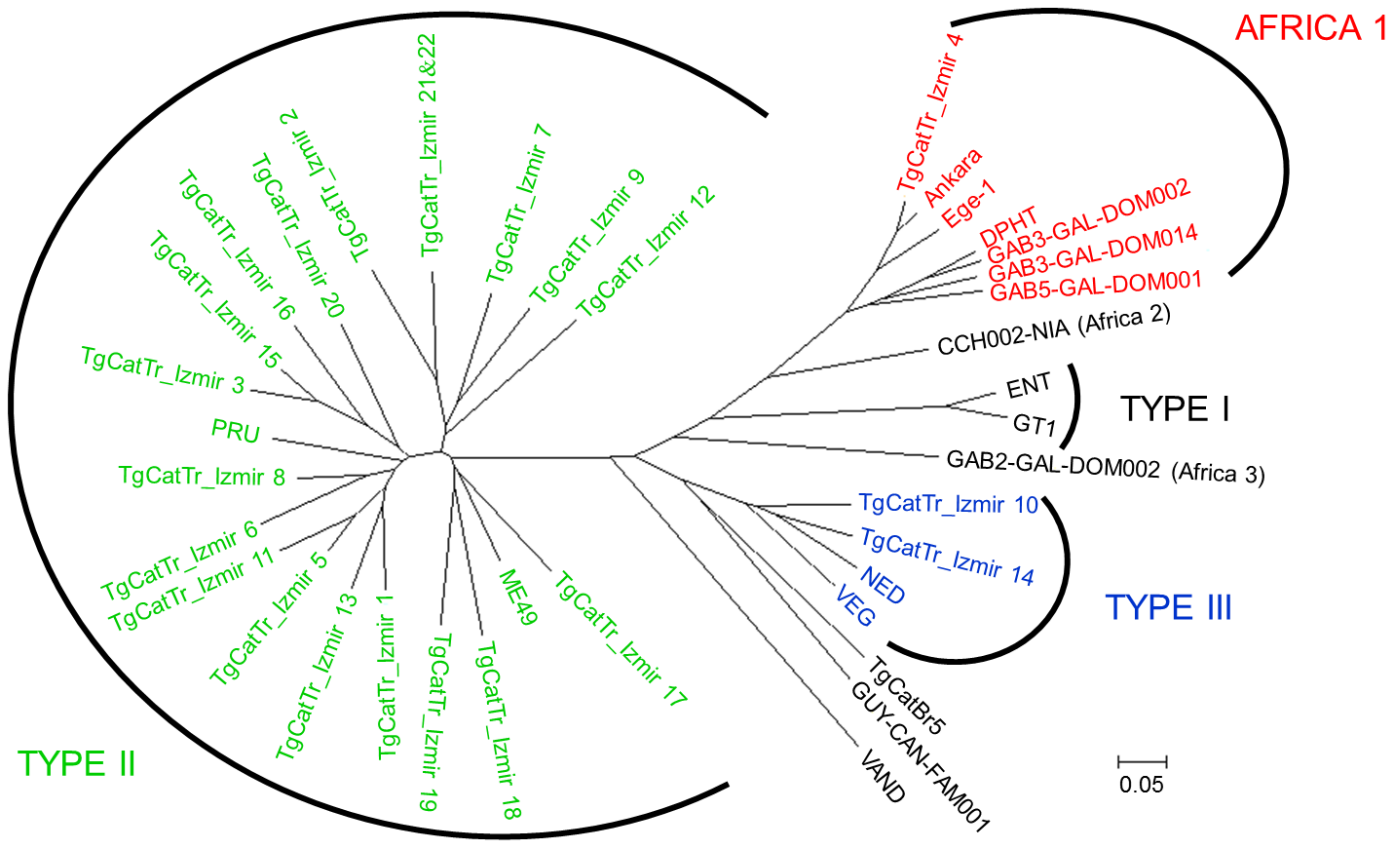
Clustering analysis of 22 strains isolated from stray cats of İzmir and 17 reference *T. gondii* strains.

Genotyping results of 22 strains according to microsatellite analysis with 15 markers are shown in table 2. Africa 1, Type II, and Type III strains were defined by eight microsatellite markers *(TUB2, W35, TgM-A, B18, B17, M33, IV.1, and XI.1)* but varying degrees of polymorphisms in other microsatellites were detected *(M48, M102, N60, N82, AA, N61, and N83)* especially in Type II strains. Cluster analysis showed that *T. gondii* strains isolated from stray cats in İzmir belong to three different groups as described above (Figure 2). Interestingly, Ankara and Ege-1 (previously isolated from congenital toxoplasmosis cases in Turkey), and TgCatTr_Izmir 4 strains are closely related isolates within Africa 1 group. The strains within Type II lineage have different genetic variability except two strains isolated from the same area (TgCatTr_Izmir 21&22) which have same genetic pattern (Figure 2, Table 1).

**Table 2:** Genotyping results with 15 microsatellite markers of the 22 strains isolated from stray cats of İzmir and 17 reference *T. gondii* strains.

ISOLATE (GENOTYPE)	Microsatellite marker (size; base pair)														
	*TUB2* (287–291)	*W35* (242–248)	*TgM-A* (203–211)	*B18* (156–170)	*B17* (334–366)	*M33* (165–173)	*IV.1* (272–282)	*XI.1* (354–362)	*M48* (209–243)	*M102* (164–196)	*N60* (132–157)	*N82* (105–145)	*AA* (251–332)	*N61* (79–123)	*N83* (306–338)
TgCatTr_Izmir 1 (Type II)	289	242	207	158	336	169	274	356	227	176	142	117	263	87	314
TgCatTr_Izmir 2 (Type II)	289	242	207	158	336	169	274	356	227	172	145	131	263	99	310
TgCatTr_Izmir 3 (Type II)	289	242	207	158	336	169	274	356	227	176	140	115	279	95	312
TgCatTr_Izmir 4 (Africa 1)	291	248	205	160	342	-	274	354	231	166	147	111	295	91	310
TgCatTr_Izmir 5 (Type II)	289	242	207	158	336	169	274	356	223	176	142	-	273	87	314
TgCatTr_Izmir 6 (Type II)	289	242	207	158	336	169	274	356	211	176	140	123	275	85	314
TgCatTr_Izmir 7 (Type II)	289	242	207	158	336	169	274	356	213	178	140	111	263	97	316
TgCatTr_Izmir 8 (Type II)	289	242	207	158	336	169	274	356	219	176	142	123	-	85	310
TgCatTr_Izmir 9 (Type II)	289	242	207	158	336	169	274	356	213	178	140	119	295	101	310
TgCatTr_Izmir 10 (Type III)	289	242	205	160	336	165	278	356	213	190	147	111	267	87	314
TgCatTr_Izmir 11 (Type II)	289	242	207	158	336	169	274	356	223	176	142	127	277	85	314
TgCatTr_Izmir 12 (Type II)	289	242	207	158	336	169	274	356	215	178	138	109	297	109	310
TgCatTr_Izmir 13 (Type II)	289	242	207	158	336	169	274	356	213	176	140	117	259	87	314
TgCatTr_Izmir 14 (Type III)	289	242	205	160	336	165	278	356	213	190	149	111	265	91	312
TgCatTr_Izmir 15 (Type II)	289	242	207	158	336	169	274	356	227	176	140	115	265	97	312
TgCatTr_Izmir 16 (Type II)	289	242	207	158	336	169	274	356	221	176	142	115	271	103	312
TgCatTr_Izmir 17 (Type II)	289	242	207	158	336	169	274	356	213	174	140	109	265	91	308
TgCatTr_Izmir 18 (Type II)	289	242	207	158	336	169	274	356	215	174	140	125	261	87	314
TgCatTr_Izmir 19 (Type II)	289	242	207	158	336	169	274	356	231	174	142	111	263	97	314
TgCatTr_Izmir 20 (Type II)	289	242	207	158	336	169	274	356	241	176	140	113	271	89	310
TgCatTr_Izmir 21 (Type II)	289	242	207	158	336	169	274	356	213	172	145	111	267	97	310
TgCatTr_Izmir 22 (Type II)	289	242	207	158	336	169	274	356	213	172	145	111	267	97	310
ANKARA (Africa 1)	291	248	205	160	342	165	274	354	227	166	147	111	295	91	310
EGE-1 (Africa 1)	291	248	205	160	342	165	274	354	227	166	149	111	289	91	310
DPHT (Africa 1)	291	248	205	160	342	165	274	354	225	166	147	111	271	89	306
GAB3-GAL-DOM014 (Africa 1)	291	248	205	160	342	165	274	354	229	166	142	111	271	95	306
GAB5-GAL-DOM001 (Africa 1)	291	248	205	160	342	165	274	354	231	166	149	111	277	87	306
GAB3-GAL-DOM002 (Africa 1)	291	248	205	160	342	165	274	354	223	166	147	111	269	89	306
CCH002-NIA (Africa 2)	289	248	205	160	336	165	274	354	225	166	145	111	273	89	308
GAB2-GAL-DOM002 (Africa 3)	291	242	207	160	342	165	278	354	223	166	142	111	277	97	310
ENT (Type I)	291	248	209	160	342	169	274	358	209	166	145	119	267	87	306
GT1 (Type I)	291	248	209	160	342	169	274	358	209	168	145	119	265	87	306
Me49 (Type II)	289	242	207	158	336	169	274	356	215	174	142	111	265	91	310
PRU (Type II)	289	242	207	158	336	169	274	356	209	176	142	117	265	123	310
NED (Type III)	289	242	205	160	336	165	278	356	209	190	147	111	267	91	312
VEG (Type III)	289	242	205	160	336	165	278	356	213	188	153	111	267	89	312
TgCatBr5 (Atypical)	291	242	205	160	362	165	278	356	237	174	140	111	265	89	314
VAND (Amazonian)	291	242	203	162	344	167	276	356	217	170	142	113	277	91	308
GUY-CAN-FAM001 (Caribbean 1)	291	242	205	162	342	165	278	356	213	164	142	109	265	87	312

### 3. Seroprevalence

The presence of anti-*T. gondii* IgG antibodies were investigated in 1121 cats (1021 healthy and 100 deceased cats) by *in house* ELISA and IFA. *In house* ELISA was optimized based on the OD results of 46 cat samples as detected by ID. VET ELISA. The ID VET ELISA results were positive in all the sera from *T. gondii* isolate-positive cats. Among the 36 sera randomly selected from the *T. gondii* isolate-negative cats, 12 (33.3%) of them were seropositive both with ID VET ELISA and in house ELISA whereas 11 (30.5%) of them were positive with IFA. After the analysis of OD values of ID VET ELISA and in house ELISA as well as IFA results, 7 negative and positive sera concordant in all assays were used as negative and positive controls during in house ELISA and IFA.

In deceased and healthy stray cats together (n: 1121), the seropositivity rate was 34.2% (384/1121) and 35.6% (400/1121) according to IFA and *in house* ELISA, respectively. Among them, the serology results of 94 samples were discordant and after these discordant samples were discarded, the seropositivity rate among those positive in both assays serology coherent samples was 33.5% (345/1027). The distribution of seropositivity rates in serology coherent samples in different locations of İzmir is shown in figure 1. The range of seroprevalence changes between 0–54.5% in which the highest seropositivity rate was detected in Güzelbahçe (Figure 1).

In deceased stray cats, the seropositivity rate was 42% (42/100) and 48% (48/100) according to IFA and *in house* ELISA, respectively. Among them, the serology results of 6 samples were discordant and after these discordants were discarded, the seropositivity rate among those positive in both assays was 44.6% (42/94).

In healthy stray cats, the seropositivity rate was 33.4% (342/1021) and 34.4% (352/1021) according to IFA and *in house* ELISA, respectively. Among them, the serology results of 88 samples were discordant and after these discordants were discarded, the seropositivity rate among those positive in both assays was 32.4% (303/933) (Table 3).

**Table 3:** Toxoplasmosis seropositivity rates among stray cats of İzmir using IFA and *in house* ELISA.

Assays	Deceased group		Healthy group		Deceased and healthy groups	
	Number of cats	Seropositivity rate (%)	Number of cats	Seropositivity rate (%)	Number of cats	Seropositivity rate (%)
*In house* ELISA	100	48	1021	34.4	1121	35.6
IFA	100	42	1021	33.4	1121	34.2
Positive in both assays a	94	44.6	933	32.4	1027	33.5

a The seropositivity rates were calculated among the sera in which the IFA and *in house* ELISA results were coherent.

Interestingly, the seropositivity rates among the deceased cats was significantly higher than healthy stray cats (*P* = 0.0033).

## Discussion

Detection of Africa 1 genotype from two congenital toxoplasmosis cases in Turkey was remarkable finding since atypical Africa 1 genotype is described in sub-Saharan Africa, especially in immunocompromised patients. In this group of patients, toxoplasmosis occurs as a reactivation of a past infection acquired in different African countries. Also, it has been isolated from domestic animals collected in Gabon [2], [6], [18]. In continental Europe, Africa 1 genotype has only been detected in a French renal transplant patient who has acquired toxoplasmosis following renal transplantation (the donor of unknown origin was seropositive and recipient seronegative) and the origin of the donor may be related to sub-Saharan Africa [2]. In Brazil, BrI genotype which seems to be similar to Africa 1 genotype has been isolated from various animals [1], [26].

The first aim of the present study is to investigate the presence of three major clonal lineages or atypical strains in İzmir which is close to historic trade routes and has a huge wild life park with bird sanctuary (Figure 1). These specifications make İzmir a suitable location to isolate various genotypes. Therefore, the present study aimed to determine the prevalent strains in stray cats of İzmir.

Among the 22 strains genotyped, Africa 1 was isolated for the first time from a stray cat in İzmir. In addition, two major clonal lineages Type II (86.3%) and Type III (9%) were also prevalent in İzmir. In Europe, Asia, Americas and Africa, three major clonal lineages Type I, II and III as well as atypical and recombinant strains exist. In France, Type II is the most prevalent strain (>95%) isolated from humans and animals [2]. In Germany, among the 68 strains isolated from cats, 83.8% were Type II and 1.4% were Type III [27]. In North America, clonal population similar to Europe is being detected [14]. In Central and South Americas, strains are characterized by their high genetic diversity [1], [28]–[30].

In Africa, the population structure of isolated strains is similar to that of Europe and Northern America with a limited genetic diversity until now [31]. Besides the archetypal genotypes, atypical strains such as Africa 1, 2, and 3 have been isolated from humans and domestic animals in West and Central Africa [2], [5], [6]. In Egypt, among the strains isolated from cats, 53% were Type II and 36.5% were Type III. In Asia, the population structure also show a limited genetic diversity [1]. In China, most of genotyping data come from China where the three major clonal lineages have been identified in addition to the predominant atypical genotype, Chinese 1 [10], [11]. Among cats in China, the Chinese 1 genotype (also designated as ToxoDB genotype #9) the three archetypal clonal lineages (I, II, and III) are the most prevalent strains [32], [33].

In the present study, two major clonal lineages Type II and Type III strains previously isolated in Europe, Asia, North America and Chile in South America were also isolated in stray cats of İzmir located on Western coast of Turkey. Overall, these findings show that major clonal lineages as well as atypical strains are being transported among continents through trade routes or long distance migratory birds.

The secondary aim of the present study was to determine toxoplasmosis seroprevalence in a large stray cat population of İzmir. The potential risk of stray cats for human toxoplasmosis can be investigated by determining toxoplasmosis seroprevalence in stray cats of İzmir, as well as whole country. Toxoplasmosis seroprevalence varies between 30–40% in domestic cats worldwide [34]. In some countries, epidemiological surveys demonstrate that antibody against *T. gondii* may be detected in up to 74% of cats in some populations, depending on their lifestyle [35], [36]. In Turkey, toxoplasmosis seropositivity rates in cats were determined locally in central (40.3% and 76% in Ankara and Niğde, respectively) and eastern (55.5% in Elazığ province) Turkey using Sabin Feldman Dye test [37]–[39]. Seropositivity rate varies between 9–46% in Germany, France, and Italy. The seropositivity rates in Central and South America vary between 42–74% and 18–73, respectively. In America, the seropositivity rate was reported to be 31.6% [18]. In Asia and Africa, seropositivity rates vary between 8–33% and 91–97%, respectively [40], [41].

In the present study, toxoplasmosis seroprevalence in stray cats of İzmir (1021 healthy and 100 deceased cats) was first time determined. Using IFA and *in house* ELISA, the seropositivity rates in deceased and healthy cats were between 42–48% and 33.4–34.4%, respectively. Previously, human toxoplasmosis seroprevalence in Aydın and İzmir provinces, located in Western Turkey, were reported as 30.1% and 49.4%, respectively [42], [43]. These results suggest stray cats as a risk factor for human toxoplasmosis in İzmir. Interestingly, seropositivity rate of deceased cat group was significantly higher than healthy cat group (*P* = 0.0033) which implies toxoplasma infection as a possible contributory factor in death among stray cats of İzmir.

Overall, two major clonal lineages (Type II, Type III) and an atypical genotype, Africa 1 have been isolated for the first time from stray cats in İzmir. Specific geographic location of Turkey between three continents, previous isolation of atypical Africa 1 genotype in two congenital toxoplasmosis cases and in a stray cat in the present study as well as predominant detection of Type II strains, frequently identified in Europe, indicates the probable transportation of these strains between continents through trade routes or long distance migratory birds. Further studies are required to isolate more strains from the wild life of Turkey because additional atypical strains may exist. İzmir has a large bird sanctuary located in a wild life park where these atypical strains may be isolated. The high seroprevalence and the variety of *T. gondii* genotypes supported the theory of, stray cats as a risk factor for human toxoplasmosis in İzmir. Future studies should assess the transmission potential of cats and wild life in different regions of Turkey.
